# Prevalence, risk factors and short-term consequences of adverse birth outcomes in Zimbabwean pregnant women: a secondary analysis of a cluster-randomized trial

**DOI:** 10.1093/ije/dyab248

**Published:** 2021-12-07

**Authors:** Bernard Chasekwa, Robert Ntozini, James A Church, Florence D Majo, Naume Tavengwa, Batsirai Mutasa, Christie Noble, Nadia Koyratty, John A Maluccio, Andrew J Prendergast, Jean H Humphrey, Laura E Smith, for the Sanitation Hygiene Infant Nutrition Efficacy (SHINE) Trial Team

**Affiliations:** Zvitambo Institute for Maternal and Child Health Research, Harare, Zimbabwe; Zvitambo Institute for Maternal and Child Health Research, Harare, Zimbabwe; Blizard Institute, Queen Mary University of London, London, UK; Zvitambo Institute for Maternal and Child Health Research, Harare, Zimbabwe; Zvitambo Institute for Maternal and Child Health Research, Harare, Zimbabwe; Zvitambo Institute for Maternal and Child Health Research, Harare, Zimbabwe; Blizard Institute, Queen Mary University of London, London, UK; Department of Population Medicine and Diagnostics, Cornell University, Ithaca, NY, USA; Department of Economics, Middlebury College, Middlebury, VT, USA; Zvitambo Institute for Maternal and Child Health Research, Harare, Zimbabwe; Blizard Institute, Queen Mary University of London, London, UK; Department of International Health, Johns Hopkins Bloomberg School of Public Health, Baltimore, MD, USA; Zvitambo Institute for Maternal and Child Health Research, Harare, Zimbabwe; Department of International Health, Johns Hopkins Bloomberg School of Public Health, Baltimore, MD, USA; Zvitambo Institute for Maternal and Child Health Research, Harare, Zimbabwe; Department of Population Medicine and Diagnostics, Cornell University, Ithaca, NY, USA

**Keywords:** Miscarriage, stillbirth, low birthweight, preterm birth, neonatal mortality, obstetric care, Zimbabwe

## Abstract

**Background:**

Globally, 15 million children are born preterm each year and 10.7 million are born at term but with low birthweight (<2500 g).

**Methods:**

The Sanitation Hygiene Infant Nutrition Efficacy (SHINE) cluster-randomized trial enrolled 5280 pregnant women between 22 November 2012 and 27 March 2015 to test the impact of improved water supply, sanitation and hygiene, and improved infant feeding, on child growth and anaemia. We conducted a secondary analysis to estimate the prevalence and risk factors of miscarriage, stillbirth, preterm birth, size small for gestational age (SGA), low birthweight (LBW), perinatal mortality, and neonatal mortality, and to estimate the effects of adverse birth outcomes on infant survival and growth.

**Results:**

The prevalence of adverse birth outcomes was: miscarriage: 5.0% [95% confidence interval (CI), 4.4, 5.7]; stillbirth: 2.3% (95% CI 1.9, 2.7); preterm birth: 18.2% (95% CI 16.9, 19.5); SGA: 16.1% (95% CI 15.0, 17.3); LBW: 9.8% (95% CI 9.0, 10.7); and neonatal mortality: 31.4/1000 live births (95% CI 26.7, 36.5). Modifiable risk factors included maternal HIV infection, anaemia, lack of antenatal care and non-institutional delivery. Preterm infants had higher neonatal mortality [risk ratio (RR): 6.1 (95% CI 4.0, 9.2)], post-neonatal infant mortality [hazard ratio (HR): 2.1 (95% CI 1.1, 4.1)] and stunting at 18 months of age [RR: 1.5 (95% CI 1.4, 1.7)] than term infants; 56% of stillbirths and 57% of neonatal deaths were among preterm births.

**Conclusions:**

Neonatal mortality and stillbirth are high in Zimbabwe and appear to be driven by high preterm birth. Interventions for primary prevention of preterm birth and strengthened management of preterm labour and ill and small neonates are required to reduce neonatal mortality in Zimbabwe and other African countries with similar profiles.

Key Messages

- Globally, 15 million children are born preterm and 10.7 million are born at term but weigh less than 2.5 kg. These two groups of infants comprise 20% of births but 50% of neonatal deaths.
- In this population-based sample of more than 5000 pregnant women and their infants in rural Zimbabwe, 2.3% (95% CI 1.9, 2.7) of pregnancies ended in stillbirth and neonatal mortality was 31.4/1000 live births (95% CI 26.7, 36.5). Among infants who provided gestational age and weight at birth, 18.2% (95% CI 16.9, 19.5) were born preterm; 57% of both stillbirths and neonatal deaths were among preterm infants.
- Neonatal mortality is high in Zimbabwe and appears to be driven by high preterm birth.
- Interventions to reduce preterm births and to strengthen pre-emptive management of preterm labour and care for small and ill neonates are urgently required to reduce neonatal mortality in Zimbabwe and other African countries with similar profiles.

## Background

Globally, 15 million infants are born preterm (<37 weeks’ gestation)^1^ every year, and 965 000 of these children die as neonates.^2^ An additional 23.3 million infants are born at term, but small for gestational age (SGA); of these, 10.7 million are low birthweight (LBW, <2500 g) and 336 800 term LBW infants die as neonates.^3^ Together, preterm and term LBW infants comprise 20% of global births, but make up 50% of the 2.6 million neonatal deaths every year. Furthermore, infants in these vulnerable groups who survive the neonatal period are at continued heightened risk of morbidity, mortality and poor growth and development.^4^^,^^5^ Nearly doubling this annual early life death toll are 2.1 million stillbirths,^6^ about half of which occur during labour. Reducing stillbirth, preterm birth and neonatal mortality (NNM) is widely considered ‘unfinished business’ of the United Nations (UN) Millennium and Sustainable Development Goals, because these deaths occur disproportionately in low-and-middle-income countries (LMICs).^7^ Indeed, 77% of stillbirths,^6^ 81% of preterm births^1^ and 79% of neonatal deaths^8^ occur in South Asia and sub-Saharan Africa alone. Finally, second trimester miscarriage (fetal death between 14 and 28 weeks’ gestation) occurs disproportionately in LMICs, with reported prevalence of 5–6%^9^^,^^10^ compared with <1% in high-income countries.^11^

Zimbabwe has stubbornly high NNM: in 2019 it was 32/1000 live births,^12^ even higher than it was 30 years earlier in 1990 (26.9/1000).^13^ A 2010 review reported that Zimbabwe had a preterm birth rate of 17%, the fourth highest in the world.^14^ A subsequent review reported a lower rate (12%), but this estimate was based on merged data across several countries in the region.^1^ In 2015, the stillbirth rate in Zimbabwe was 30.5/1000 births, which was unchanged from 1980.^6^

The Sanitation Hygiene Infant Nutrition Efficacy (SHINE) trial was a community-based study in two rural districts (Shurugwi and Chirumanzu, Midlands Province) of Zimbabwe which enrolled 5280 pregnant women at a median gestational age of 12 weeks [interquartile range (IQR) 9–16, min = 1.6, max = 42.1} and followed their infants to 18 months postpartum. Households in these districts primarily subsist through small-scale farming and poultry and cattle keeping. The area is sparsely populated (18.6 people per km^2^)^15^ with few paved roads, limited public transportation and virtually no electric grid or piped water. Elevation in the area is ∼1400 m, and both districts are categorized as pre-elimination, non-malaria endemic.^16^ Elsewhere we report that urogenital schistosomiasis was detected in 26.8% of SHINE women, but was not associated with miscarriage, stillbirth, preterm birth, SGA or neonatal death.^17^ The objectives of the current analyses were to estimate the prevalence of miscarriage, stillbirth, preterm birth, SGA, LBW and NNM in the SHINE cohort, identify risk factors and estimate the neonatal, perinatal and infant mortality and child stunting and wasting at 18 months associated with preterm birth, SGA and LBW. We hypothesized that a deeper understanding of the burden of adverse birth outcomes and their associated determinants and mortality risk may shed light on what actions should be prioritized to reduce neonatal mortality in Zimbabwe.

## Methods

The Medical Research Council of Zimbabwe and the Institutional Review Board of the Johns Hopkins Bloomberg School of Public Health reviewed and approved the study protocol.

The SHINE trial has been described previously^18^ and primary outcomes reported.^19^^,^^20^ In brief, SHINE was a cluster-randomized community-based trial testing the independent and combined effects of infant and young child feeding (IYCF) and household water, sanitation and hygiene (WASH) on length-for-age Z score (LAZ) of children at 18 months of age; see [https://osf.io/w93hy] for protocol and analysis plan. Between 22 November 2012 and 27 March 2015, village health workers (VHWs) employed by the Zimbabwe Ministry of Health and Child Care (MoHCC) surveyed women 15–49 years of age, living in two rural districts, every 5 weeks to identify those who had missed a menstrual period and to offer urine pregnancy testing. Eligible pregnant women were referred to SHINE; research nurses obtained written informed consent before enrolling them into the trial. We initially limited recruitment to women who were <14 weeks’ gestation, to provide time to construct a latrine, and two handwashing stations, and to deliver soap and behaviour change communication promoting use of these facilities in the WASH households prior to birth.

However, over the recruitment period, the cut-off of gestational age for recruitment eligibility was liberalized to <18 weeks, <24 weeks and finally no gestational age cut-off, to maximize recruitment. Women residing in the study area temporarily, as employees, or in rented premises (i.e. situations where latrine construction would be outside the woman’s authority) were also ineligible. Gestational age was based on maternal history of last menstrual period. Ultrasound dating of pregnancies was not available in the study districts. Within 2 weeks of consent, research nurses collected baseline household socioeconomic status data,^21–22^ measured maternal height, weight, mid-upper arm circumference, blood pressure and haemoglobin concentration (Hemocue, Angelholm, Sweden), and tested mothers for HIV using a rapid test algorithm. Maternal capabilities, defined as psychosocial characteristics that determine women’s child- and self-care capacity,^23–25^ were also assessed. These included: depression (score ≥12 on the Edinburgh postnatal depression scale and/or suicidal ideation, a definition validated among Zimbabwean women^26^); perceived health status, decision-making autonomy, mothering self-efficacy, gender norm attitudes; perceived social support; and perceived time stress. Higher scores depicted poorer capability for depression (more depressive symptoms) and perceived time stress (higher levels of time stress), but greater capability for perceived health status (better health), decision making autonomy (more autonomous decision-making), mothering self-efficacy (greater mothering self-efficacy) and perceived social support (greater social support).

Women were referred to the local MoHCC clinic for antenatal care if they had not already booked their pregnancy. At the time of SHINE, the MoHCC guidelines for antenatal care included at least four antenatal clinic visits, daily iron-folate supplementation, urinalysis, syphilis testing, vaginal examination and blood pressure measurement. Diagnosed morbidities were treated according to national guidelines. Prevention of mother-to-child HIV transmission guidance changed from World Health Organization (WHO) Option B [maternal antiretroviral therapy (ART) from 14 gestational weeks until the end of breastfeeding] to Option B+ (lifelong ART for all pregnant and breastfeeding women) in November 2013. The MoHCC also provides ‘waiting women’s shelters’ at health facilities to accommodate women near the end of their pregnancy, to facilitate institutional delivery. Infant birth date, weight and delivery details were transcribed from health facility records. The trial provided Tanita BD-590 infant scales to all health institutions in the study area and trained facility staff on weighing newborns at birth. Recumbent length was measured to the nearest 0.1 cm using a Seca 417 infantometer (Weigh and Measure LLC, Olney, MD) and weight to the nearest 10 g (Tanita BD-590 infant scale) at 18 months of age. At the 18-month trial endpoint, mothers and infants were visited anywhere in the country. However, in view of the household-based nature of the WASH interventions, intermediate visits were done only when the mother was available in the household where she consented.

Serious adverse events, including fetal loss (miscarriages or stillbirths) and neonatal deaths, were ascertained by research nurses or VHWs or reported to the study by the mother herself; a senior nurse collected event details which were reviewed by the study physician (A.J.P.) before reporting to institutional review boards (IRBs). Adverse birth outcomes were defined as: miscarriage (fetal death before 28 weeks’ gestation); stillbirth (fetal death ≥28th weeks’ gestation); preterm birth (live birth before 37 weeks’ gestation); LBW (birthweight <2500 g); SGA (<10th percentile weight for gestational age using INTERGROWTH standards^27^); perinatal death (stillbirth or infant death within 8 days of postnatal age, with the birth day being taken as Day 0); and neonatal death (infant death before 28 days of age).

The Medical Research Council of Zimbabwe and the Institutional Review Board of the Johns Hopkins Bloomberg School of Public Health approved the SHINE study protocol. All participants provided written informed consent. The trial protocol is available at https://osf.io/w93hy.

### Statistical methods

Prevalence for each adverse birth outcome was calculated; 95% confidence intervals (CI) were estimated using a bootstrap resampling technique with 1000 bootstraps.^28^ Values of gestational age at delivery of live births were considered plausible if ≥24 weeks and ≤42 weeks and 6 days, and where birthweight-for-gestational age was ≥0.4th centile and ≤99.6th centile using INTERGROWTH references.^29^ The primary estimate of preterm birth was based on infants who provided birthweight and plausible gestational age; two sensitivity estimates were also conducted which included: (i) infants in the primary analysis plus infants missing birthweight; and (ii) infants in the primary analysis and those missing birthweight, plus infants missing gestational age assuming their preterm birth prevalence was equivalent to those in the primary estimate.

Infant lengths and weights at 18 months were converted to Z scores using WHO reference standards; stunting and wasting were defined as LAZ <−2 and WLZ <−2, respectively.^30^ Maternal mid-upper arm circumference was categorized as: <23 cm (underweight), 23−<27 cm (normal weight), 27−<31cm (overweight) and ≥31 cm (obese), which corresponded to body mass index (BMI) of, respectively, <18.5, 18.5−<25, 25−<30 and ≥30 kg/m^2^ in a validation study among pregnant women in South Africa who were <20 weeks’ gestation.^31^

To identify risk factors for adverse birth outcomes, we pre-specified two groups of variables. Group 1 included factors reported in previous epidemiological and clinical studies. For all outcomes, these included maternal age (modelled at <20, 20 <35, ≥35 years), height, mid-upper arm circumference, HIV infection and anaemia (Hb <12 g/dL; haemoglobin was adjusted for trimester of pregnancy and elevation, given the relatively high elevation in the area^32^), hypertension (>140 mmHg systolic OR >90 mmHg diastolic), receipt of antenatal care, either maternal education or household wealth (these were highly collinear, so the one more strongly associated in univariable analyses with the pregnancy outcome was offered to its multivariable model), twin/triplet pregnancy (except for miscarriage), place of delivery and infant sex (for live birth outcomes). Group 2 included other factors measured in SHINE: household food insecurity^22^ and water and sanitation services, marital status and all of the maternal capabilities. The unadjusted relative risk of each birth outcome associated with each exposure factor was estimated in univariable models. In adjusted analyses, backward elimination regression was implemented forcing retention of trial arm and all Group 1 variables, and setting retention of Group 2 factors at p <0.1; retained variables were then modelled with generalized estimating equations (GEE) to adjust for clustering. We employed multiple imputation by chained equations to account for any missing data, assuming a missing‐at‐random pattern (Stata MICE software). For perinatal and neonatal mortality outcomes, two adjusted models were constructed, each including one of the two four-group classifications: preterm/term with LBW/normal birthweight (NBW) or preterm/term with SGA/appropriate for gestational age (AGA).

We calculated neonatal and post-neonatal infant mortality and child stunting and wasting at 18 months according to weight and gestational age at birth categories. We also calculated the relative risk of these outcomes (except for post-neonatal infant mortality), where the reference category was Term, NBW, AGA, Term/NBW, or Term/AGA, as relevant. Hazard ratios for post-neonatal mortality for these categories were estimated using Cox proportional hazard model with standard errors adjusted for the clusters. All statistical analyses were performed using STATA version 14.^33^

## Results

**Figure 1:**
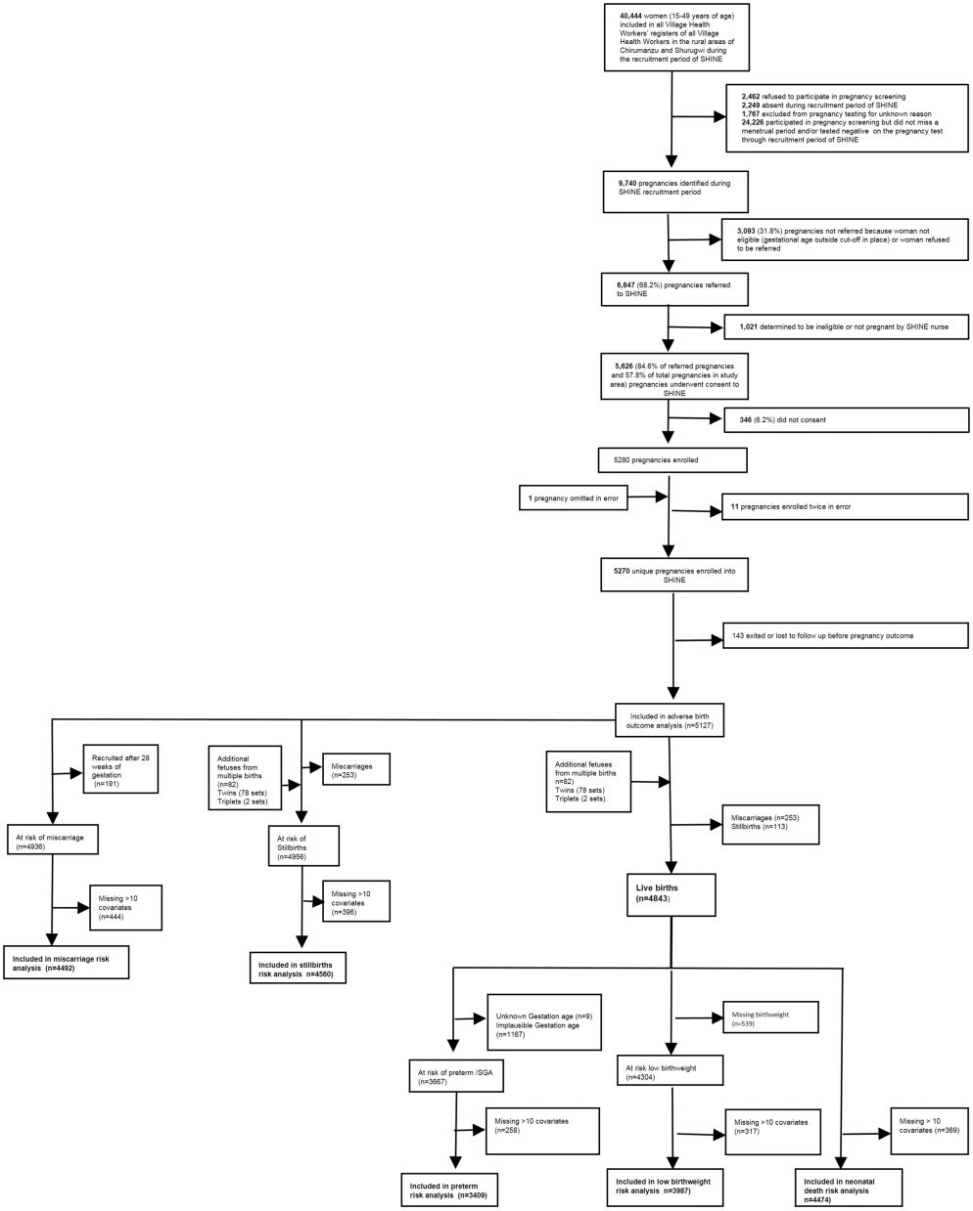
Flow diagram of participants

Over the recruitment period, the MoHCC VHWs recorded a total of 40 444 women aged 15–49 years as residing in the two study districts (Figure 1). Of these, 6478 (16.0%) women were either absent during VHW visits or refused pregnancy testing, and 24 226 had not missed a menstrual period or tested negative on a pregnancy test at all VHW visits. Of the 9740 pregnancies identified, 3093 (31.8%) were not referred to SHINE because the pregnancy was beyond the gestational age cut-off in place at the time of identification, the woman met another exclusion criterion or the woman refused to be referred to SHINE. Of the remaining 6647 pregnancies referred to SHINE by VHWs: 1021 were found to be ineligible by data clerk or by the research nurse; 346 (6.2%) of the 5626 eligible women did not consent; and 5280 (79.5% of referred pregnancies and 54% of all pregnancies) were enrolled. After correction for enrolment errors and exclusion of 143 women who exited the study with unknown birth outcomes, 5127 pregnant women with known birth outcomes were included in the current analyses. Women missing ≥10 exposure variables (most of whom were not available in their home for the baseline visit when this information was collected) were included in prevalence estimates but dropped from risk factor analyses.

At baseline women were, on average, 26.3 years old, and had completed more than 9 years of schooling; 95.2% were married; 15.9% were living with HIV; and 20.1% were anaemic (Table 1). Nearly all women booked for antenatal care and 87.8% delivered in a health care facility. Mean infant weight and gestational age at birth were 3.1 kg and 37.3 weeks, respectively.

**Table 1:** Baseline maternal, household, antenatal and delivery, and infant factors^a^

	*N* = 5127
**Maternal factors (*n*)**	
Age (y) (SD)	26.3 (6.7) [4457]
Gestational age at recruitment, wk, median (IQR)	12.0 (9.4, 15.6) [5102]
Parity, median (IQR),	2 (1, 3) [3255]
Height (cm) (SD)	160.1 (5.9) [4850]
Mid-upper arm circumference (cm) (SD)	26.4 (3.1) [4891]
Married	95.2 (4460/4685)
Completed schooling (y) (SD)	9.6 (1.8) [4720]
Religion	
Apostolic Christian	43.3 (2219/5127)
Other Christian	41.0 (2104/5127)
Other religion	7.8 (399/5127)
Missing religion	7.9 (404/5127)
HIV-infected	15.9 (779/5127)
Haemoglobin (g/dL) (SD)	12.0 (1.5) [4100]
Anaemic	20.1 (820/4070)
*S. haematobium*, microscopy positive	10.6 (468/4397)
Haematuria positive	24.5 (1043/4264)
Consumes minimally diverse diet	39.5 (1784/4518)
**Household factors (*n*)**	
Number of occupants (SD)	4.9 (2.2) [4777]
Has a latrine	36.3 (1658) [4564]
Members practice open defecation	55.4 (2583) [4665]
Feces observed in yard	30.9 (1418) [4594]
Drinking water from improved source	63.0 (2886) [4582]
Walk time to water source (min) median (IQR)	10 (5, 20) [4560]
**Antenatal and delivery factors (*n*)**	
Ever booked for antenatal care	97.6 (4605/4718)
Institutional delivery	87.8 (3796/4324)
Vaginal delivery	92.7 (4069/4391)
**Infant factors at birth (live births) (*n*)**	n = 4843
Birthweight (kg) (SD)	3.08 (0.49) [4304]
Gestational age (weeks) (SD)	37.3 (6.3) [4834]
Male	50.4 (2430/4817)
Twin or triplet	2.9 (142/4843)

SD, standard deviation; IQR, interquartile range; y, years; wks, weeks.

a Maternal and household characteristics presented for mothers who had a known birth outcome; baseline data collected about 2 weeks after recruitment; maternal antenatal and delivery characteristics transcribed from health records or by maternal history during postnatal visits.

### Prevalence and risk factors of adverse birth outcomes

Among 4936 women enrolled into the trial before 28 weeks’ gestation, 249 [5.0% (95% CI 4.4, 5.7)] miscarried (Supplementary Table S1, available as Supplementary data at *IJE* online). Maternal age ≥35 years was the only independent risk factor in the adjusted model. There were 158 [3.2% (95% CI 2.8, 3.8)] second trimester miscarriages; risk factors were similar when analyses excluded first trimester miscarriages (data not shown). The prevalence of stillbirth was 2.3% (95% CI 1.9, 2.7) (Supplementary Table S1, available as Supplementary data at *IJE* online). Antenatal care, singleton pregnancy, improved latrine ownership (prior to the trial) and maternal anaemia were protective against stillbirth. Notably, 63/113 (56%) of the stillbirths occurred before 37 weeks’ gestation (data not shown). Perinatal mortality was 47.0/1000 births (95% CI 41.4, 53.3) (Supplementary Table S1, available as Supplementary data at *IJE* online). Compared with infants born at term with normal birthweight, risk of perinatal mortality was higher for infants born small or prematurely and highest for infants born small and prematurely, where small size was based on either SGA or LBW (Supplementary Table S1, available as Supplementary data at *IJE* online).

Among the 4843 live births, birthweight and plausible gestational age at birth were available for 3667 (75%); among these infants, 666 (18.2%, 95% CI 16.9, 19.5) were preterm births (Table 2). In the adjusted model, maternal age ≥35 years, anaemia, unmarried status, shorter stature, multiple fetus pregnancy and male infant sex were associated with greater risk of preterm birth, and maternal overweight and obesity were protective. 

**Table 2:** Prevalence and risk factors for preterm birth, small for gestational age and low birthweight among women enrolled in SHINE trial (2012–17)^a^

	Preterm		Small for gestational age				Low birthweight		
	18.2% (95% CI 16.9, 19.5)		16.1 % (95% CI 15.0, 17.3)				9.8% (95% CI 9.0, 10.7)		
	[666/3667]		[591/3667]				[422/4304]		
	Unadj. RR (95% CI)	Adj. RR (95% CI)	Unadj. RR (95% CI)		Adj. RR (95% CI)		Unadj. RR (95% CI)		Adj. RR (95% CI)
	*n *=* *3409	*n *=* *3030	*n = *3409		*n *=* *2892		*n *=* *3987		*n *=* *3303
**Maternal factors**						
Age, y						
<20	1.17 (0.98, 1.40)	1.01 (0.81, 1.26)	**1.39 (1.17, 1.65)**	**1.44 (1.16, 1.79)**	**1.46 (1.17, 1.82)**	**1.41 (1.07, 1.85)**
20–35	1.00	1.00	1.00	1.00	1.00	1.00
>35	**1.36 (1.10, 1.68)**	**1.46 (1.14, 1.87)**	0.89 (0.67, 1.17)	0.95 (0.70, 1.30)	1.30 (0.97, 1.74)	1.34 (0.95, 1.90)
Married	**0.48 (0.37, 0.61)**	**0.51 (0.37, 0.70)**	0.93 (0.63, 1.39)	Not retained	**0.59 (0.41, 0.85)**	Not retained
Education, y	**0.94 (0.91, 0.98)**	0.97 (0.93, 1.02)	1.01 (0.97, 1.06)	1.01 (0.96, 1.07)	0.96 (0.91, 1.01)	0.97 (0.92, 1.04)
Maternal height	**0.99 (0.99, 1.00)**	**0.99 (0.98, 1.00)**	**0.99 (0.98, 0.99)**	**0.99 (0.98, 1.00)**	**0.98 (0.98, 0.99)**	**0.98 (0.98, 0.99)**
Maternal MUAC, cm						
<23 (thin)	1.17 (0.94, 1.46)	1.12 (0.86, 1.47)	**1.49 (1.20, 1.84)**	**1.38 (1.05, 1.82)**	**1.81 (1.42, 2.32)**	**1.98 (1.47, 2.67)**
23 <27 (normal)	1.00	1.00	1.00	1.00	1.00	1.00
27 <31(overweight)	**0.70 (0.58, 0.84)**	**0.66 (0.53, 0.82)**	**0.81 (0.67, 0.98)**	0.83 (0.66, 1.04)	**0.69 (0.53, 0.89)**	**0.62 (0.45, 0.84)**
≥31 (obese)	**0.62 (0.43, 0.87)**	**0.65 (0.44, 0.95)**	**0.54 (0.36, 0.81)**	0.68 (0.44, 1.05)	0.71 (0.46, 1.09)	0.71 (0.41, 1.22)
HIV-infected	1.08 (0.89, 1.31)	0.93 (0.73, 1.18)	**1.25 (1.03, 1.52)**	**1.31 (1.02, 1.67)**	**1.46 (1.16, 1.83)**	1.32 (0.98, 1.77)
Anaemic	**1.40 (1.18, 1.67)**	**1.26 (1.01, 1.56)**	1.09 (0.89, 1.32)	0.95 (0.75, 1.22)	**1.30 (1.03 1.65)**	1.07 (0.80, 1.43)
Hypertensive	0.95 (0.58, 1.57)	1.06 (0.61, 1.84)	1.14 (0.70, 1.84)	1.02 (0.56, 1.86)	1.17 (0.65, 2.11)	1.24 (0.63, 2.44)
Maternal capabilities^b^						
Depression	1.28 (0.97, 1.69)	Not retained	0.94 (0.67, 1.33)	Not retained	0.76 (0.48, 1.20)	0.72 (0.41, 1.26)
Low PHS	0.89 (0.76, 1.04)	Not retained	0.97 (0.82, 1.13)	Not retained	0.89 (0.73, 1.08)	0.96 (0.76, 1.21)
Low DMA	1.02 (0.87, 1.18)	Not retained	1.02 (0.87, 1.20)	Not retained	1.00 (0.82, 1.23)	Not retained
Low MSE	0.98 (0.84, 1.14)	Not retained	1.140.9 (0.98, 1.34)	Not retained	1.04 (0.86, 1.26)	Not retained
Low GNA	**1.18 (1.02, 1.38)**	Not retained	1.12 (0.95, 1.32)	Not retained	1.09 (0.89, 1.34)	Not retained
Low PSS	0.93 (0.80, 1.08)	0.87 (0.73, 1.04)	1.07 (0.92, 1.25)	Not retained	1.06 (0.88, 1.29)	0.91 (0.72, 1.15)
Low PTS	1.00 (0.86, 1.16)	Not retained	1.09 (0.94, 1.27)	Not retained	1.16 (0.96, 1.41)	Not retained
**Household factors**						
Wealth score^c^	1.01 (0.97, 1.06)	Not retained	0.97 (0.93, 1.01)	Not retained	0.98 (0.93, 1.03)	Not retained
Food insecurity^d^	1.07 (0.89, 1.29)	Not retained	1.03 (0.84, 1.25)	Not retained	1.15 (0.91, 1.45)	Not Retained
No improved latrine	1.11 (0.95, 1.31)	Not retained	0.91 (0.77, 1.07)	Not retained	0.88 (0.72, 1.07)	0.89 (0.70, 1.13)
Feces in yard	**1.17 (1.01, 1.37)**	1.18 (0.99, 1.41)	0.92 (0.78, 1.09)	Not retained	1.09 (0.89, 1.34)	Not retained
Improved drinking water	0.90 (0.77, 1.05)	Not retained	0.93 (0.79, 1.09)	Not retained	**0.78 (0.64, 0.94)**	0.84 (0.67, 1.06)
**Antenatal/delivery factors**						
Booked ANC	0.47 (0.19, 1.17)	0.46 (0.15, 1.46)	0.63 (0.19, 2.05)	0.54 (0.14, 2.12)	0.62 (0.17, 2.26)	0.48 (0.12, 1.95)
Non-inst delivery	Not applicable	Not applicable	1.12 (0.84, 1.50)	1.25 (0.91, 1.73)	**1.77 (1.33, 2.36)**	**1.98 (1.43, 2.75)**
Twin/triplet	**3.24 (2.66, 3.94)**	**3.06 (2.26, 4.14)**	**2.87 (2.27, 3.62)**	**3.25 (2.32, 4.55)**	**7.00 (5.84, 8.39)**	**8.09 (5.98, 10.96)**
Hungry season delivery^c^	1.02 (0.88, 1.18)	1.04 (0.88, 1.23)	1.07 (0.92, 1.25)	1.09 (0.91, 1.30)	1.20 (0.99, 1.45)	1.15 (0.92, 1.45)
Female infant	**0.84 (0.72, 0.97)**	**0.83 (0.70, 0.99)**	1.07 (0.92, 1.25)	1.16 (0.97, 1.39)	1.18 (0.98, 1.43)	**1.30 (1.04, 1.64)**
	*n *=* *3409		*n = *3409		*n *=* *3983	
**Study arm^e^**						
SOC	1.00	1.00	1.00	1.00	1.00	
IYCF	0.84 (0.66, 1.07)	0.84 (0.65, 1.09)	1.09 (0.88, 1.35)	1.15 (0.90, 1.46)	1.00 (0.75, 1.33)	1.01 (0.75, 1.37)
WASH	0.99 (0.79, 1.24)	0.97 (0.76, 1.23)	1.14 (0.93, 1.40)	1.10 (0.86, 1.39)	1.12 (0.85, 1.47)	0.98 (0.72, 1.31)
WASH+IYCF	0.98 (0.79, 1.23)	0.99 (0.78, 1.26)	1.02 (0.83, 1.26)	1.08 (0.85, 1.36)	0.99 (0.75, 1.31)	0.91 (0.67, 1.22)

a RR, risk ratio; LB, live births; Small for gestational age, <10th centile weight for gestational age using Intergrowth Fetal Growth Standards; low birthweight, <2500 g at birth; anaemic, haemoglobin concentration during pregnancy <12 µg/dl; HTN, hypertensive during pregnancy defined as systolic blood pressure >140 mm Hg and/or diastolic blood pressure >90 mm Hg; MUAC, mid-upper arm circumference; ANC, antenatal care. Maternal and household baseline data were collected about 2 weeks after consent was recorded (at roughly 14 weeks’ gestation). This gap created opportunity for loss to follow-up between consent and baseline; thus for all outcomes, the number of mothers included in the risk factor analysis is less than the denominator used to calculate prevalence. Bold indicates factors asssociated at P value <0.05.

b For further discussion of all maternal capabilities see reference.^20^ Low PHS, low perceived health status defined as mother perceives herself to have poor health status; low DMA, low decision-making autonomy defined as mother perceives herself to have little decision-making autonomy; low MSE, low mothering self-efficacy, mother perceives herself not to be efficacious in her mothering skills; low GNA, low gender norm attitudes defined as mother holds inequitable gender norm attitudes; low PSS, low perceived social support defined as mother perceives herself to have little social support; low PTS, low perceived time stress defined as mother perceives herself as unstressed.

c Hungry season is period of relative food scarcity defined as October–March.

d Preterm birth defined as <37 weeks’ gestation calculated from last menstrual period.

e Study arms of SHINE trial. SOC, Standard of Care; IYCF, Infant and Young Child Feeding; WASH, Water And Sanitation, Hygiene; WASH+IYCF, WASH and IYCF interventions delivery concurrently.

Among the 3667 infants with available birthweight and gestational age data, 591 (16.1%, 95% CI 15.0, 17.3) were SGA at birth (Table 2). In adjusted analyses, mothers who were <20 years of age, underweight, shorter, carrying a twin/triplet pregnancy, and HIV-positive were at greater risk for delivering an SGA infant, and mothers who were overweight or obese were at reduced risk of SGA.

Of the 4304 infants who provided birthweight, nearly 10% were LBW (9.8%, 95% CI 9.0, 10.7) (Table 2). In adjusted analyses, maternal age <20 years, shorter stature, thinness, multiple fetus pregnancy, non-institutional delivery and female infant sex were independent risk factors; maternal overweight was protective against LBW.

There were 152 neonatal deaths among the 4843 live births (31.4/1000, 95% CI 26.9, 36.5) (Table 3). In the adjusted analysis, only maternal anaemia, term LBW and preterm LBW were independent risk factors.

**Table 3:** Prevalence and risk factors of neonatal mortality among women enrolled in SHINE trial (2012–17**)^a^**

	Neonatal death31.4/1000 LBW (95% CI 26.9, 36.5)(152/4843)		
	Unadj RR (95% CI)	Adj RR (95% CI)	Adj RR (95% CI)
	*n *=* *4461	*n *=* *2982	*n *=* *2982
		LBW model	SGA model
**Maternal factors**			
Age			
<20 years	**1.53 (1.03, 2.25)**	1.42 (0.72, 2.80)	1.65 (0.86, 3.18)
20–35, years	1.00	1.00	1.00
>35, years	**1.84 (1.17, 2.88)**	1.10 (0.48, 2.52)	1.29 (0.57, 2.91)
Married	0.98 (0.44, 2.21)	–	**–**
Education, years	**0.91 (0.84, 0.99)**	0.98 (0.84, 1.13)	0.97 (0.84, 1.11)
Height	0.99 (0.98, 1.01)	1.00 (0.97, 1.03)	0.99 (0.97, 1.01)
MUAC, cm			
<23 (thin)	0.95 (0.61, 1.48)	0.87 (0.35, 2.14)	0.95 (0.38, 2.32)
23 <27 (normal)	1.00	1.00	1.00
27 <31(overweight)	0.72 (0.51, 1.00)	0.91 (0.40, 2.06)	0.70 (0.32, 1.55)
≥31 (obese)	0.88 (0.52, 1.48)	2.29 (0.94, 5.58)	2.40 (0.99, 5.80)
HIV-infected	1.21 (0.80, 1.84)	0.67 (0.29, 1.56)	0.77 (0.33, 1.76)
Anaemic	**1.72 (1.17, 2.53)**	**1.98 (1.02, 3.90)**	1.65 (0.85, 3.20)
Hypertensive	0.66 (0.17, 2.59)	–	–
Maternal capabilities^b^			
Depression	0.56 (0.23, 1.35)	–	
Low PHS	0.70 (0.50, 1.00)	0.73 (0.41, 1.32)	0.72 (0.41, 1.28)
Low DMA	0.87 (0.62, 1.23)	–	
Low MSE	0.97 (0.68, 1.36)	–	
Low GNA	1.08 (0.76, 1.52)	–	
Low PSS	0.85 (0.61, 1.18)	–	
Low PTS	1.14 (0.82, 1.58)	–	
**Household factors**			
Wealth score^c^	0.96 (0.87, 1.05)	–	
Food insecurity^d^	1.18 (0.79, 1.77)	–	
No improved latrine	1.15 (0.80, 1.65)	–	
Feces in yard	0.94 (0.66, 1.34)	**–**	
Improved drinking water	1.25 (0.87, 1.78)	–	
**Antenatal/delivery factors**			
Booked ANC	0.40 (0.10, 1.51**)**	–	
Non-institutional delivery	**2.09 (1.32, 3.33)**	1.44 (0.62, 3.36)	1.55 (0.68, 3.51)
Twin/triplet	**4.95 (3.15, 7.77)**	1.59 (0.69, 3.67)	**3.23 (1.44, 7.25)**
Hungry season delivery^e^	1.01 (0.73, 1.39)	0.86 (0.49, 1.50)	0.87 (0.50, 1.51)
Female infant	**0.70 (0.50, 0.98)**	0.93 (0.53, 1.63)	1.03 (0.60, 1.78)
Infant birthweight, kg^f^	**0.17 (0.13, 0.21)**	**–**	
Preterm birth	**5.99 (3.90, 9.20)**	**–**	
Small for gestational age	1.11 (0.63, 1.94)	**–**	
**Term NBW**	**1.00** ^5^	**1.00**	
Preterm NBW	**2.15 (1.08, 4.24)**	1.49 (0.55, 4.05)	
Term LBW	**3.10 (1.22, 7.91)**	**4.69 (1.70, 12.98)**	
Preterm LBW	**18.31 (11.56, 29.02)**	**17.05 (8.57, 33.91)**	
**Term AGA**	**1.00e**	**–**	**1.00**
Term SGA	1.70 (0.82, 3.51)	–	2.02 (0.85, 4.80)
Preterm AGA	**6.49 (4.00, 10.53)**	**–**	**5.25 (2.72, 10.13)**
Preterm SGA	**10.56 (4.27, 26.17)**	**–**	**7.19 (2.08, 24.76)**
**Study arm** ^g^			
SOC	1.00	1.00	1.00
IYCF	1.12 (0.66, 1.92)	0.84 (0.37, 1.91)	0.89 (0.39, 2.03)
WASH	1.25 (0.75, 2.08)	0.89 (0.42, 1.90)	0.94 (0.44, 1.99)
WASH+IYCF	1.24 (0.75, 2.06)	1.04 (0.50, 2.13)	1.17 (0.56, 2.42)

a RR, risk ratio; LB, live births; MUAC, mid-upper arm circumference; ANC, antenatal care. SGA, small for gestational age defined as <10th centile weight for gestational age using Intergrowth Fetal Growth Standards; LBW, low birthweight defined as weight <2500 g at birth; NBW, normal birthweight; anaemic: haemoglobin concentration <12 µg/dl; Hypertensive: systolic blood pressure >140 mm Hg and/or diastolic blood pressure >90 mm Hg; preterm birth: <37 weeks’ gestation calculated from last menstrual period. Maternal and household baseline data were collected about 2 weeks after consent was recorded (at roughly 14 weeks’ gestation). This gap created opportunity for loss to follow-up between consent and baseline; thus, for all outcomes, the number of mothers included in the risk factor analysis is less than the denominator used to calculate prevalence. Bold indicates factors associated at P value <0.05.

b For further discussion of all maternal capabilities see Matare.^1^ Low PHS, low perceived health status defined as mother perceives herself to have poor health status; low DMA, low decision-making autonomy defined as mother perceives herself to have little decision-making autonomy; low MSE, low mothering self-efficacy, mother perceives herself not to be efficacious in her mothering skills; low GNA, low gender norm attitudes defined as mother holds inequitable gender norm attitudes; low PSS, low perceived social support defined as mother perceives herself to have little social support; low PTS, low perceived time stress defined as mother perceives herself as unstressed.

c Wealth score was an asset index created for SHINE^2^.

d Food insecurity defined as Coping Strategy Index.^3^

e Hungry season is period of relative food scarcity defined as October–March.

f Infant birthweight, preterm status and small for gestational age status were entered into univariate analyses. In adjusted analyses, these variables were not entered singly but as part of Term/Preterm/NBW/LBW category.

g Study arms of SHINE trial. SOC, Standard of Care; IYCF, Infant and Young Child Feeding; WASH, Water And Sanitation, Hygiene; WASH+IYCF, WASH and IYCF interventions delivery concurrently.

1 Matare C, Mbuya M, Pelto G, Dickin K, Maluccio J, Stoltzfus R. Assessing maternal capabilities in the SHINE Trial: a heretofore overlooked piece in the causal pathways to child health. *Clin Infect Dis* 2015;**61(Suppl 7)**:S745–51).

2 Chasekwa B, Maluccio JA, Ntozini R *et al.* Measuring wealth in rural communities: lessons from the Sanitation, Hygiene, Infant Nutrition Efficacy (SHINE) trial. *PLoS One* 2018;**13**:e0199393.

3 Maxwell D, Watkins B, Wheeler R, Collins G. The Coping Strategy Index: A tool for rapid measurement of household food security and the impact of food aid programs in humanitarian emergencies. Nairobi: CARE and WFP, 2003.

### Sensitivity estimates of preterm birth

As reported above, our primary estimate of preterm birth, based on the 3667 (75% of live births) infants who provided birthweight and a plausible gestational age, was 18.2% (95% CI 16.9, 19.5). Infants excluded from this estimate included 539 infants missing birthweight and 637 missing gestational age (missing or implausible last menstrual period). Of the 539 infants missing birthweight, 186 (34.5%, 95% CI 30.5, 38.7) were born preterm based on last menstrual period. To interpret this high preterm birth rate, we analysed place of delivery and neonatal mortality for all live births and the three subgroups of infants (Table 4). Compared with all live births, a greater proportion of the 3667 infants with complete data were born in a health institution and neonatal mortality was substantially lower (Table 4). Among infants missing birthweight, fewer had an institutional delivery and NNM was 81.6/1000 live births (Table 4). For the remaining 637 infants missing gestational age, place of delivery and NNM were similar to the total population. Sensitivity analyses yielded preterm birth estimates of 20.3% (95% CI 19.1, 21.6) and 20.0% (18.9, 21.0), respectively (Table 4).

**Table 4:** Sensitivity estimates of preterm birth among infants participating in the SHINE trial

Subgroups of live births	*N*	Health institution delivery *n* (%)	Neonatal mortality (95% CI)	Preterm prevalence (95% CI) *n*
(1) Birthweight and plausible gestational age available	3667	3399 (92.7%)	23.5 (18.7, 30.0)	18.2 (16.9, 19.5) 666
(2) Missing birthweight	539	149 (27.6%)^a^	81.6 (62.7, 110.3)	34.5 (30.5, 38.7) 186
(3) Gestational age missing or implausible	637	575 (90.3%)	31.4 (20.4, 48.1)	
Total live births	4843	4252 (87.8%)	1. (26.7, 36.5)	
Groups 1 + 2				20.3 (19.1, 21.6)
Groups 1 + 2 + 3, assuming preterm prevalence of Group 3 ≈ Group 1				20.0 (18.9, 21.0)

^a^Place of delivery was health institution (149), home (204), and unknown (186).

### NNM, post-neonatal infant mortality and stunting associated with preterm birth and small size at birth

Infant survival and growth outcomes associated with gestational age and size at birth were analysed among the 3667 infants with complete data. NNM was higher among preterm compared with term births [risk ratio (RR) 6.1, 95% CI: 4.0, 9.2]; preterm infants comprised 57% of the neonatal deaths. Similarly, the NNM was higher among LBW compared with NBW (RR 9.8, 95% CI 6.6, 14.6). Among infants who were both preterm and LBW, NNM was of 201.1/1000 Live births: these infants comprised just 5% of the population but contributed 42.5% of the neonatal deaths. Similarly, NNM among all SGA infants was 20% greater than among all AGA infants and was highest among infants with both preterm birth and SGA (Table 5).

**Table 5:** Risk of neonatal and post-neonatal infant mortality and of child stunting and wasting at 18 months of age according to gestational age and weight at birth categories^a^

Birth outcome	Neonatal mortality			Post-neonatal infant mortality			Child stunting at 18 months			Child wasting at 18 months		
	*n/N*	(95% CI)	RR (95% CI)	*n/N*	(95% CI)	HR (95% CI)	*n/N*	% (95% CI)	RR (95% CI)	*n/N*	% (95% CI)	RR (95% CI)
Term	37/3001	12.3 (9.0, 17.0)	1.0	30/2964	10.0 (7.2, 14.1)	1.0	837/2878	29.1 (27.4, 30.8)	1.0	81/2865	2.8 (2.3, 3.5)	1.0
Preterm	50/666	75.1 (57.6, 97.2)	6.1 (4.0, 9.2)	13/616	22.7 (13.4, 38.4)	2.1 (1.1, 4.1)	261/583	44.8 (40.8, 48.8)	1.5 (1.4, 1.7)	17/578	2.9 (1.9, 4.6)	1.0 (0.6, 1.7)
≥2500 g	44/3333	13.2 (9.9, 17.5)	1.0	34/3289	10.6 (7.6, 14.8)	1.0	938/3183	29.4 (27.9, 31.1)	1.0	83/3165	2.6 (2.1, 3.2)	1.0
<2500 g	43/334	128.7 (96.7, 169.4)	9.8 (6.5, 14.6)	9/291	30.9 (16.0, 58.8)	3.3 (1.6, 7.0)	160/278	57.6 (51.6, 63.3)	2.0 (1.7, 2.2)	15/278	5.4 (3.3, 8.7)	2.1 (1.2, 3.5)
AGA	71/3076	23.1 (18.3, 29.1)	1.0	34/3005	11.6 (8.4, 16.1)	1.0	831/2908	28.6 (26.9, 30.3)	1.0	69/2892	2.4 (1.9, 3.0)	1.0
SGA	16/591	27.1 (16.7, 43.5)	1.2 (0.7, 2.0)	9/575	15.6 (8.3, 29.4)	1.5 (0.7, 3.2)	267/553	48.3 (44.2, 52.3)	1.7 (1.5, 1.9)	29/551	5.3 (3.7, 7.4)	2.2 (1.4, 3.4)
Term NBW	31/2851	10.9 (7.7, 15.3)	1.0	26/2820	9.2 (6.2, 13.8)	1.0	763/2739	27.9 (26.1, 29.6)	1.0	73/2726	2.7 (2.1, 3.4)	1.0
Preterm NBW	13/482	27.0 (16.0, 45.1)	2.5 (1.3, 4.7)	9/469	19.2 (9.9, 36.8)	1.8 (0.8, 4.1)	175/444	39.4 (35.0, 44.0)	1.4 (1.2, 1.6)	10/439	2.3 (1.2, 4.2)	0.9 (0.4, 1.6)
Term LBW	6/150	40.0 (18.1, 86.2)	3.7 (1.6, 8.7)	4/144	27.8 (10.6, 70.8)	3.3 (1.1, 9.5)	74/139	53.2 (44.9, 61.4)	1.9 (1.6, 2.2)	8/139	5.8 (2.9, 11.1)	2.1 (1.1, 4.4)
Preterm LBW	37/184	201.1 (148.2, 266.9)	18.5 (11.8, 29.1)	5/147	34.0 (14.6, 77.3)	4.1 (1.5, 10.7)	86/139	61.9 (53.9, 69.3)	2.2 (1.9, 2.6)	7/139	5.0 (2.4, 10.2)	1.9 (0.9, 4.0)
Term LBW	6/150	40.0 (18.1, 86.2)	1.0	4/144	27.8 (10.6, 70.8)	1.0	74/139	53.2 (44.9, 61.4)	1.0	8/139	5.8 (2.9, 11.1)	1.0
Preterm LBW	37/184	201.1 (148.2, 266.9)	5.0 (2.2, 11.6)	5/147	34.0 (14.6, 77.3)	1.2 (0.3, 4.6)	86/139	61.9 (53.9, 69.3)	1.2 (0.9, 1.4)	7/139	5.0 (2.4, 10.2)	0.9 (0.3, 2.3)
Preterm NBW	13/482	27.0 (16.0, 45.1)	1.0	9/469	19.2 (9.9, 36.8)	1.0	175/444	39.4 (35.0, 44.0)	1.0	10/439	2.3 (1.2, 4.2)	1.0
Preterm LBW	37/184	201.1 (148.2, 266.9)	7.5 (4.1, 13.7)	5/147	34.0 (14.6, 77.3)	2.3 (0.7, 7.2)	86/139	61.9 (53.9, 69.3)	1.6 (1.3, 1.9)	7/139	5.0 (2.4, 10.2)	2.2 (0.9, 5.7)
AGA Term	26/2453	10.6 (7.1, 15.7)	1.0	21/2427	8.7 (5.6, 13.4)	1.0	595/2363	25.2 (23.5, 27.0)	1.0	56/2352	2.4 (1.8, 3.1)	1.0
SGA Term	11/548	20.1 (11.3, 35.4)	1.9 (0.9, 3.8)	9/537	16.8 (8.6, 32.5)	2.1 (1.0, 4.7)	242/515	47.0 (42.6, 51.4)	1.9 (1.7, 2.1)	25/513	4.9 (3.3, 7.1)	2.0 (1.3, 3.2)
AGA Preterm	45/623	72.2 (54.6, 94.9)	6.8 (4.2, 11.0)	14/578	24.2 (14.4, 40.4)	2.7 (1.3, 5.5)	236/545	43.3 (39.1, 47.5)	1.7 (1.5, 1.9)	13/540	2.4 (1.4, 4.1)	1.0 (0.6, 1.8)
SGA Preterm	5/43	116.3 (48.9, 251.2)	11.0 (4.4, 27.2)	0/38	–	–	25/38	65.8 (49.7, 78.9)	2.6 (2.1, 3.3)	4/38	10.5 (4.2, 23.9)	4.4 (1.7, 11.6)

a RR, risk ratio; HR, hazard ratio; SGA, small for gestational age defined as <10th centile weight for gestational age using Intergrowth Fetal Growth Standards; AGA, appropriate for gestational age; LBW, low birthweight defined as weight <2500 g at birth; NBW, normal birthweight.

Preterm, LBW and SGA infants who survived the neonatal period remained at increased risk of death in the post-neonatal period of infancy (Table 5). Stunting was prevalent among all SHINE children: even among term normal birthweight infants, 27.9% were stunted at 18 months. However, term LBW, pretermNBW and preter -LBW were 1.4 to 2.2 times more likely to be stunted compared with term normal birthweight children.

## Discussion

The high neonatal mortality among infants in the SHINE cohort is comparable to the national average estimated in 2019 (31.4/1000 and 32/1000 live births,^12^ respectively), and both estimates are higher than the rate reported by the World Health Organization for Zimbabwe 30 years earlier in 1990 (26.9/1000).^13^ This high NNM has persisted despite the Zimbabwe MoHCC achieving high coverage of prevention of mother-to-child transmission (PMTCT) (94% in 2018),^34^ antenatal care (93% with ≥1 visit and 74% with ≥4 visits) and institutional delivery (88%).^12^

Based on our findings from SHINE, we posit that Zimbabwe’s stubbornly high neonatal mortality is largely driven by a high rate of preterm births. In our primary analysis, the preterm birth prevalence was 18.2%, one of the highest in the world.^1^^,^^14^ However, we believe our sensitivity estimate of 20% is closer to the true preterm birth prevalence in SHINE. This sensitivity estimate included 539 infants who did not provide a birthweight, primarily because they were born outside a health institution. Among these infants, the preterm birth prevalence was 35%, which was plausibly consistent with their high neonatal mortality (81.6/1000).

Preterm birth has been universally resistant to change. Globally, the prevalence of low birthweight declined from 17.5% to 14.6 between 2000 and 2015 %,^35^ whereas the preterm birth rate increased from 9.8% to 10.6%.^1^ Thus, most of the progress in birthweight has been mediated through improved intra-uterine growth and reduced SGA, which is more responsive than preterm birth to the improvements in maternal nutritional status^36^ that occurred over this period.^37^ Accordingly, whereas about 35% of neonatal deaths worldwide are due to preterm birth—primarily reflecting contexts like the large countries in South Asia where SGA prevalence remains much higher than preterm birth—in the SHINE cohort, the preterm birth prevalence exceeded SGA prevalence (18% vs 16%) and 57% of the neonatal deaths were among infants born preterm. This typology of perinatal morbidity and mortality is not unique to Zimbabwe; in several countries in the region, NNM is high and preterm birth rates approximate or exceed SGA rates. For example, 2012 rates of NNM, PT and SGA were, respectively, 26.8/1000 live births, 12.3% and 10.1% in Kenya, and 26.9/1000 live births, 18.1%, and 15.1% in Malawi.^3^

Our study has several limitations. First, this was a secondary analysis of a cluster-randomized trial whose primary objective was to test the impact of WASH and IYCF on children at 18 months of age. The pregnancy surveillance undertaken to identify women early in pregnancy resulted in many women booking for antenatal care earlier than they would have otherwise, which hastened initiation on PMTCT interventions for HIV-positive women and may have resulted in earlier detection and treatment of other problems. Beyond this, the trial neither provided enhanced antenatal care nor did it assess the quality of antenatal care. Second, about one-third of the pregnancies identified by VHWs were not referred to SHINE, providing opportunity for selection bias. The main reason for non-referral was ineligibility due to gestational age beyond the cut-off in place at the time. Since nulliparous women have a tendency for higher preterm birth and SGA deliveries than most multiparous women,^38^ we calculated the proportion of nulliparous women enrolled during periods of a different exclusion criterion. We found that when enrolment was restricted to pregnancies of gestational age <14, <18, <24 weeks and <parturition, the proportion of nulliparous women enrolled was, respectively, 9.4%, 11.9%, 16.9% and 22.5%. Thus SHINE likely under-represented nulliparous women, and adverse birth rates in the SHINE study sample may be an under-estimate of those in the source population. Third, though the study districts were chosen for the SHINE trial because they had levels of child stunting, sanitation coverage and livelihoods similar to most other rural districts in Zimbabwe, they were not statistically representative of the whole country; the prevalence of adverse birth outcomes is not strictly generalizable to the country as a whole. However, our estimates are based on a large sample size, and neonatal mortality within the SHINE cohort was nearly the same as the national estimate from the 2019 Multiple Indicator Cluster Survey,^12^ suggesting that our estimates are likely to be similar to those of the whole country. Fourth, fetal ultrasound and newborn examinations were not available to more accurately estimate gestational age at delivery; we did, however, limit our primary analyses to infants with plausible weight for gestational age. Finally, pre-pregnancy and post-delivery maternal weight were not measured, so gestational weight gain, an important determinant of adverse birth outcomes, was not available.

In the past two decades, there has been a great deal of progress in improving clinical management of infants with preterm birth in LMICs, with strategies including antenatal corticosteroids, maternal and neonatal antibiotics, neonatal stabilization and resuscitation and prevention of hypothermia.^39^ However, these strategies require institutional delivery. Our sensitivity analyses suggest that a substantial proportion of infants with preterm birth in rural Zimbabwe may be born outside a health institution, where they would not benefit from these intrapartum and immediate newborn care strategies. Therefore, in addition to scaling up clinical management, evidence-based interventions for primary prevention of preterm labour will also be required.^40^ Interventions that address psychosocial stress may be important^41^ and are being piloted.^42^ Pharmaceutical interventions that show promise are high-dose calcium (which is recommended by WHO to prevent preeclampsia but is also associated with a 24% reduction in preterm birth^43^); low-dose aspirin (recommended for decades specifically for high risk pregnancies but recently shown in the ASPIRIN trial of 12 000 women in six LMICs to be safe and effective in reducing preterm birth by 11%)^44^; and multiple micronutrients (MMS) which include iron-folate, in lieu of iron-folate only (compared with iron-folate, MMS reduced preterm birth by 11%, when initiated before 20 weeks’ gestation^45^). Zimbabwean women may particularly benefit from these interventions because their diet is among the lowest in micronutrient density of any country worldwide.^46^ These interventions are safe and low-cost but do present operational barriers (e.g. high-dose calcium must be taken in a divided doses 3 or 4 times daily—a requirement known to be associated with low adherence; and all three interventions are most effective when initiated earlier in pregnancy than many women initiate ANC). Implementation science studies have already been done^47–50^ and more are under way to learn how to overcome these obstacles. The combination of this added knowledge, together with continued advocacy to build political will for reducing preterm birth, will be crucial to preventing these early deaths.

Data underlying this article from the SHINE trial are available in ClinEpiDB at [http://ClinEpiDB.org].

## Supplementary Data

Supplementary data are available at *IJE* online.

## Supplementary Material

dyab248_Supplementary_Data
