# Supplementary material for: Prevalence, risk factors and short-term consequences of adverse birth outcomes in Zimbabwean pregnant women: a secondary analysis of a cluster-randomized trial
Source: Int J Epidemiol. 2021 Dec 7;51(6):1785–99. doi: 10.1093/ije/dyab248 (PMC9749708; doi:10.1093/ije/dyab248)
Supplement: dyab248_Supplementary_Data [file dyab248_supplementary_data.docx]

**Supplementary Table. Prevalence and risk factor for miscarriage, stillbirth, and perinatal mortality among women enrolled in SHINE trial (2012-2017)^1^**

|  | **Miscarriage**  **5.0% (95% CI: 4.4, 5.7)**  **[249/4936]** | | **Stillbirth**  **2.3% (95%CI: 1.9, 2.7)**  **[113/4956]** | | **Perinatal death**  **47.0/1000 Births (95% CI: 41.4, 53.3)**  **[233/4956]** | | |
| --- | --- | --- | --- | --- | --- | --- | --- |
| **Maternal Factors** | **Unadj RR**  **(95% CI)**  ***n* = 4492** | **Adj RR**  **(95% CI)**  ***n* = 4221** | **Unadj RR**  **(95% CI)**  ***n* = 4560** | **Adj RR**  **(95% CI)**  ***n* = 3905** | **Unadj RR**  **(95% CI)**  ***n* = 4956** | **Adj RR**  **(95% CI)**  ***n* = 2990**  **LBW model** | **Adj RR**  **(95% CI)**  ***n* = 2990**  **SGA model** |
| Age  <20, years  20-35, years  >35, years | 1.09 (0.72, 1.63)  1.00  **2.22 (1.51, 3.25)** | 1.11 (0.71, 1.74)  1.00  **2.29 (1.53, 3.43)** | 0.92 (0.55, 1.55)  1.00  1.39 (0.80, 2.43) | 1.06 (0.59, 1.93)  1.00  1.51 (0.82, 2.77) | 1.16 (0.83, 1.61)  1.00  1.36 (0.92, 2.00) | 1.47 (0.72, 2.98)  1.00  0.60 (0.22, 1.65) | 1.51 (0.76, 2.98)  1.00  0.74 (0.28, 1.99) |
| Married | 0.86 (0.41, 1.81) | Not retained | 2.04 (0.52, 8.06) | Not retained | 1.09 (0.55, 2.17) | - | - |
| Education, years | 0.96 (0.89, 1.05) | 0.99 (0.91, 1.09) | 1.04 (0.93, 1.16) | 1.05 (0.92, 1.20) | 0.98 (0.91, 1.05) | 0.99 (0.85, 1.15) | 1.00 (0.86, 1.16) |
| Height, cm | 0.99 (0.97, 1.02) | 1.00 (0.97, 1.03) | **0.98 (0.97, 1.00)** | 0.99 (0.97, 1.02) | **0.99 (0.98, 1.00)** | 1.00 (0.97, 1.04) | 1.00 (0.97, 1.04) |
| MUAC, cm |  |  |  |  |  |  |  |
| <23 (thin) | 0.90 (0.51,1.59) | 1.05 (0.58, 1.90) | 0.88 (0.44, 1.76) | 1.15 (0.53, 2.47) | 0.95 (0.61, 1.48) | 1.07 (0.42, 2.69) | 1.17 (0.47, 2.90) |
| 23<27 (normal) | 1.00 | 1.00 | 1.00 | 1.00 | 1.00 | 1.00 | 1.00 |
| 27<31(overweight) | 1.11 (0.79, 1.59) | 1.08 (0.73, 1.59) | 0.87 (0.42, 2.00) | 0.87 (0.51, 1.47) | 0.72 (0.51, 1.00) | 1.32 (0.60, 2.88) | 0.95 (0.45, 1.97) |
| ≥31 (obese) | 1.32 (0.77, 2.26) | 1.13 (0.64, 1.98) | 0.92 (0.42, 2.00) | 0.79 (0.31, 1.98) | 0.88 (0.52, 1.48) | 1.43 (0.46, 4.41) | 1.52 (0.50, 4.59) |
| HIV-positive | 1.39 (0.95, 2.03) | 1.36 (0.90, 2.06) | 1.43 (0.89, 2.31) | 1.44 (0.80, 2.57) | 1.25 (0.90, 1.75) | 0.84 (0.35, 1.99) | 0.86 (0.37, 2.04) |
| Anaemic | 1.05 (0.69, 1.58) | 0.96 (0.61, 1.52) | 0.73 (0.41, 1.29) | **0.48 (0.23, 0.98)** | 1.03 (0.73, 1.46) | 1.01 (0.45, 2.24) | 1.01 (0.48, 2.12) |
| Hypertensive | 1.98 (0.95, 4.13) | 1.86 (0.83, 4.14) | 1.92 (0.71, 5.19) | 1.76 (0.55, 5.60) | 1.32 (0.60, 2.91) | - |  |
| ***Maternal capabilities^2^*** | |  |  |  |  |  |  |
| Depression | 0.91 (0.47, 1.76) | Not retained | 0.80 (0.33, 1.94) | Not retained | 0.78 (0.42, 1.44) | - |  |
| Low PHS | 1.11 (0.78, 1.59) | Not retained | 1.07 (0.69, 1.64) | Not retained | 0.83 (0.63, 1.09) | 0.62 (0.31, 1.25) | 0.64 (0.33, 1.24) |
| Low DMA | 1.02 (0.74, 1.40) | Not retained | 0.89 (0.60, 1.33) | Not retained | 0.78 (0.59, 1.04) | - |  |
| Low MSE | 0.88 (0.64, 1.23) | Not retained | 1.28 (0.86, 1.90) | Not retained | 1.13 (0.86, 1.48) | - |  |
| Low GNA | **1.38 (1.01, 1.89)** | Not retained | 0.88 (0.57, 1.35) | Not retained | 1.00 (0.75, 1.33) | - |  |
| Low PSS | 1.15 (0.84, 1.57) | Not retained | 0.84 (0.56, 1.25) | 0.65 (0.41, 1.03) | 0.78 (0.59, 1.02) | - |  |
| Low PTS | 0.83 (0.61, 1.13) | Not retained | 1.22 (0.82, 1.82) | Not retained | 1.18 (0.90, 1.54) | - |  |
| **Household Factors** |  |  |  |  |  |  |  |
| Wealth score^3^ | 0.95 (0.87,1.03) | - | 1.01 (0.90, 1.12) | - | 0.98 (0.91, 1.05) | - |  |
| Food Insecurity^4^ | 1.02 (0.69, 1.52) | Not retained | 0.68 (0.38, 1.22) | 0.49 (0.24, 1.02) | 0.90 (0.63, 1.28) | - |  |
| No Improved latrine | 0.93 (0.67, 1.30) | Not retained | 1.26 (0.81, 1.98) | **1.94 (1.11, 3.38)** | 1.09 (0.81, 1.45) | - |  |
| Feces in yard | 1.06 (0.76, 1.47) | Not retained | 0.74 (0.47, 1.17) | Not retained | 0.74 (0.54, 1.01) | - |  |
| Improved drinking water | 1.16 (0.83, 1.62) | Not retained | 1.05 (0.69, 1.60) | Not retained | 1.17 (0.88, 1.56) | - |  |
| **Antenatal/delivery**  **Factors** |  | Not retained |  |  |  |  |  |
| Booked ANC | - | - | **0.19 (0.06, 0.55)** | **0.19 (0.05, 0.73)** | **0.31 (0.12, 0.77)** | - |  |
| Non-institutional delivery | - | - | **-** | **-** | **2.14 (1.46, 3.14)** | 1.76 (0.78, 3.98) | 2.02 (0.95, 4.32) |
| Twin/triplet | - | - | **4.65 (2.65, 8.16)** | **5.79 (3.11, 10.8)** | **4.79 (3.35, 6.84)** | **3.39 (1.58, 7.23)** | **6.78 (3.35, 13.70)** |
| Hungry season delivery^5^ | 1.30 (0.94, 1.79) | 1.26 (0.91, 1.75) | 1.45 (0.96, 2.18) | 1.26 (0.80, 1.99) | 1.20 (0.92, 1.57) | 1.13 (0.62, 2.06) | 1.05 (0.59, 1.88) |
| Female infant |  |  |  |  | **0.59 (0.43, 0.80)** | 0.93 (0.52, 1.67) | 1.03 (0.59, 1.82) |
| Infant birth weight, Kg^6^ |  |  |  |  | **0.15 (0.11, 0.19)** | **-** |  |
| Preterm birth |  |  |  |  | **6.07 (3.81, 9.66)** | **-** |  |
| Small for gestational age |  |  |  |  | 1.31 (0.74, 2.31) | **-** |  |
| **Term NBW** |  |  |  |  | **1.00^5^** | **1.00** |  |
| Preterm NBW |  |  |  |  | 1.64 (0.71, 3.75) | 1.25 (0.42, 3.76) |  |
| Term LBW |  |  |  |  | **3.64 (1.40, 9.45)** | **3.78 (1.31, 10.90)** |  |
| Preterm LBW |  |  |  |  | **19.71 (11.96, 32.49)** | **14.36 (6.81, 30.31)** |  |
| **Term AGA** |  |  |  |  | **1.00^5^** |  | **1.00** |
| Term SGA |  |  |  |  | 2.08 (0.98, 4.41) |  | 1.73 (0.71, 4.23) |
| Preterm AGA |  |  |  |  | **6.88 (4.01, 11.78)** |  | **4.54 (2.25, 9.16)** |
| Preterm SGA |  |  |  |  | **12.92 (5.11, 32.68)** |  | **4.93 (1.43, 17.01)** |
| **Study Arm^6^** |  |  |  |  |  |  |  |
| SOC | 1.00 | 1.00 | 1.00 | 1.00 | 1.00 | 1.00 | 1.00 |
| IYCF | 0.96 (0.58, 1.60) | 1.15 (0.68, 1.95) | **2.60 (1.37, 4.96)** | **2.17 (1.07, 4.41)** | 1.68 (1.09, 2.60) | 1.09 (0.47, 2.55) | 1.23 (0.53, 2.83) |
| WASH | 0.99 (0.61, 1.62) | 1.01 (0.60, 1.71) | 1.56 (0.78, 3.11) | 1.74 (0.84, 3.56) | 1.36 (0.87, 2.13) | 0.67 (0.27, 1.64) | 0.77 (0.32, 1.86) |
| WASH+IYCF | 1.19 (0.75, 1.90) | 1.25 (0.76, 2.06) | 1.25 (0.61, 2.55) | 1.22 (0.57, 2.63) | 1.32 (0.85, 2.07) | 1.18 (0.52, 2.63) | 1.35 (0.61, 3.00) |

^1^ RR, Risk Ratio; MUAC, Mid-upper arm circumference; SGA, Small for gestational age defined as <10^th^ centile weight for gestational age using Intergrowth Fetal Growth Standards; AGA, appropriate for gestational age; LBW, Low birth weight, weight<2500 g at birth; NBW, Normal birth weight; Anemic, hemoglobin concentration during pregnancy <12 µg/dl; Hypertensive during pregnancy defined as systolic blood pressure >140 mm Hg and/or diastolic blood pressure >90 mm Hg; ANC, antenatal care. Maternal and household baseline data were collected about 2 weeks after consent was recorded (at roughly 14 weeks’ gestation). This gap created opportunity for loss to follow-up between consent and baseline; thus, for all outcomes, the number of mothers included in the risk factor analysis is less than the denominator used to calculate prevalence.

^2^ For further discussion of all maternal capabilities see Matare, et al^1^. Low PHS, Low perceived health status defined as mother perceives herself to have poor health status ; Low DMA, Low decision-making autonomy defined as mother perceives herself to have little decision making autonomy; Low MSE, Low mothering self-efficacy, mother perceives herself not to be efficacious in her mothering skills; Low GNA, Low gender norm attitudes defined as mother holds inequitable gender norm attitudes; Low PSS, Low perceived social support defined as mother perceives herself to have little social support; Low PTS, Low perceived time stress defined as mother perceives herself as unstressed.

^3^ Wealth score was an asset index created for SHINE^2^

^4^ Food insecurity defined as Coping Strategy Index^3^

^5^ Hungry season is period of relative food scarcity defined as November – March.

^6^ Study arms of SHINE trial. SOC, Standard of Care; IYCF, Infant and young child feeding; WASH, water and sanitation, hygiene; WASH+IYCF, WASH and IYCF interventions delivery concurrently.

1. Matare CR, Mbuya MN, Pelto G, Dickin KL, Stoltzfus RJ. Assessing maternal capabilities in the SHINE trial: highlighting a hidden link in the causal pathway to child health. *Clinical Infectious Diseases* 2015; **61**(suppl_7): S745-S51.

2. Chasekwa B, Maluccio JA, Ntozini R, et al. Measuring wealth in rural communities: lessons from the Sanitation, Hygiene, Infant Nutrition Efficacy (SHINE) trial. *PLoS One* 2018; **13**(6): e0199393.

3. Maxwell D, Watkins B, Wheeler R, Collins G. The Coping Strategy Index: A tool for rapid measurement of household food security and the impact of food aid programs in humanitarian emergencies.'. *CARE and WFP, Nairobi* 2003.
